# The fauna of aquatic invertebrates in the river impacted by wastewaters from the pulp and paper industry (Komi Republic)

**DOI:** 10.3897/BDJ.9.e75362

**Published:** 2021-11-17

**Authors:** Maria Baturina, Olga Kononova, Elena Fefilova, Olga Loskutova

**Affiliations:** 1 Institute of Biology of Komi Science Centre of the Ural Branch of the Russian Academy of Sciences, Syktyvkar, Russia Institute of Biology of Komi Science Centre of the Ural Branch of the Russian Academy of Sciences Syktyvkar Russia

**Keywords:** sampling event, benthos and plankton invertebrates, Oligochaeta, Cladocera, Copepoda, Rotifera, Ephemeroptera, Plecoptera, Trichoptera, wastewaters, pulp and paper industry, Vychegda River, Komi Republic

## Abstract

**Background:**

Invertebrates are important elements of aquatic ecosystems and play a crucial role in the transformation of matter and energy in continental water bodies. Communities of aquatic invertebrates are characterised by high sensitivity to pollution by nutrients and toxic substances and acidification of water bodies; they serve as good bioindicators of the quality of the aquatic environment and impacts on hydroecosystems. All hydrobionts participate in the processes of self-purification of water bodies.

The presented dataset provides information on the aquatic invertebrate community of a large northern river. During 2018-2020, we collected data on changes in the quantitative indicators of the development of benthic and planktonic communities, as well as the species diversity of their fauna. The dataset combines information about the occurrence and abundance of benthic and planktonic invertebrates and summarises data of aquatic invertebrate species found in the Vychegda River in the zone of influence from the pulp and paper mill.

**New information:**

The presented dataset is part of a monitoring programme of the river ecosystems in the production area of Mondi Syktyvkar JSC (the European North-East of Russia, Komi Republic). The dataset describes the structure of benthic invertebrate and plankton communities in the Northern Dvina River Basin. The data on the finding and abundance of large taxa of aquatic invertebrates and species of some groups: Oligochaeta, Cladocera, Copepoda, Rotifera, Ephemeroptera, Plecoptera and Trichoptera are presented. In total, the resource includes 8720 findings of invertebrates, of which 6041 are for zoobenthos organisms and 2679 for zooplankton organisms.

## Introduction

Freshwater ecosystems are complex, dynamic and diverse. However, they are more vulnerable than marine and terrestrial environments and their biodiversity is declining much faster (Darwall 2018, Schmidt-Kloiber et al. 2019). Currently, the main factor causingglobal environmental problems, such as a reduction in biological diversity, destruction of biotopes, climate change and rapid spread of alien species, is the anthropogenic impact (Alimov et al. 2009). At the same time, there is an inverse correlation between water quality and biodiversity: to maintain a satisfactory water quality, it is necessary to maintain functionally active biodiversity of aquatic ecosystems (Ostroumov 2002).

For freshwater environmental monitoring and biodiversity simulation, data on the structure of communities are collected. Pooled species distribution data are now becoming an important (and sometimes primary) source of information in biodiversity research. However, they are often likely to be incomplete. Data gaps for a number of geographic areas and taxonomic groups, including aquatic invertebrates, exist and complicate access to solve the problems associated with global ecological and biogeographic analyses (Balian et al. 2008, Gaiji et al. 2009). Integration of data on findings of taxa is an important objective to biodiversity studies and improving prediction of future environmental changes (Shashkov et al. 2017).

It is known (Culp et al. 2000, Baturina and Kononova 2021) that wastewater from pulp and paper mills can negatively affect aquatic organisms and communities, changing their composition and structure, as well as the biology of certain species. The Vychegda (Northern Dvina River Basin) is one of thelargest rivers in the European North-East of Russia; by the Basin area (the catchment size is 121,000 km^2^), it is the second largest river in the Komi Republic. Taking into account its crucial role in the region, monitoring the state of the aquatic organisms inhabiting it is very important. After a prolonged large-scale modernisation of the manufacturing facilities of the Mondi Syktyvkar JSC and a reconstruction of the sewage treatment facilities, observations of the state of the river communities acquired the monitoring character – they were carried out at permanent posts from 2018 to 2020 as part of a joint project of the Mondi Syktyvkar JSC and the Institute of Biology of Komi Scientific Center of the Ural Branch of the Russian Academy of Sciences.

The presented dataset (Baturina et al. 2021b) provides information on the current state of diversity and species richness of the Vychegda River in the zone affected by large industrial production and can serve as a basis for water quality monitoring. It is associated with scientific articles (Baturina et al. 2021, Kononova 2021, Baturina et al. 2021a, Kononova 2021) and is an important study on the taxonomic diversity and abundance dynamics of aquatic organisms in the zone of active manufacturing, using the example of a pulp and paper mill. There is an opinion (Fefilova et al. 2021) that the fauna of aquatic invertebrates in the Russian part of the Arctic is poorly studied.

The information presented in this work enriches the fundamental knowledge about the composition of zooplankton and zoobenthos in northern rivers. This information is important both for assessing regional resources in order to carry out nature conservation measures and for studying the biogeography of freshwater invertebrates, for a detailed and in-depth analysis of the distribution of certain species or groups and for determining their ranges and ecology.

## General description

### Purpose

The main purpose of the work was to prepare a dataset on sample occurrences of zoobenthos and zooplankton invertebrates in the zone impacted by wastewaters from the pulp and paper industry (Komi Republic, Vychegda River). For datasets on sample occurrences, data from 2018 to 2020 were included and, during the period of monitor observations, a basis was prepared for assessing and analysing changes in: (1) quantitative indicators of the development of zoobenthos and zooplankton communities and (2) species diversity of aquatic invertebrate fauna, under the impact of wastewaters from the pulp and paper industry (Komi Republic, Vychegda River).

## Project description

### Title

"Russia 2021"

Distribution, systematics and spatial organisation of the aquatic invertebrate fauna in a northern river impacted by wastewaters from the pulp and paper industry (Komi Republic, Vychegda River).

Assessment of long-term impact of paper mill industry on the biological diversity in the production area.

### Study area description

All material was collected in the European part of Russia, Komi Republic: Vychegda River

Vychegda is the largest right tributary of the Northern Dvina River. It flows through the territory of the Komi Republic and the Arkhangelsk Oblast (). The length of the river is 1,130 km, the catchment area is 121,000 km^2^. Vychegda is a typical lowland river. The average density of the river network in the Vychegda River basin is 0.62 km/km^2^. Vychegda belongs to rivers with incomplete meandering. Its discharge ranges from 162 to 601 m^3^/s. In the river bed of the Vychegda River, the top (346 km long), middle (489 km long) and bottom (296 km long) parts are distinguished.

**Figure 1.**
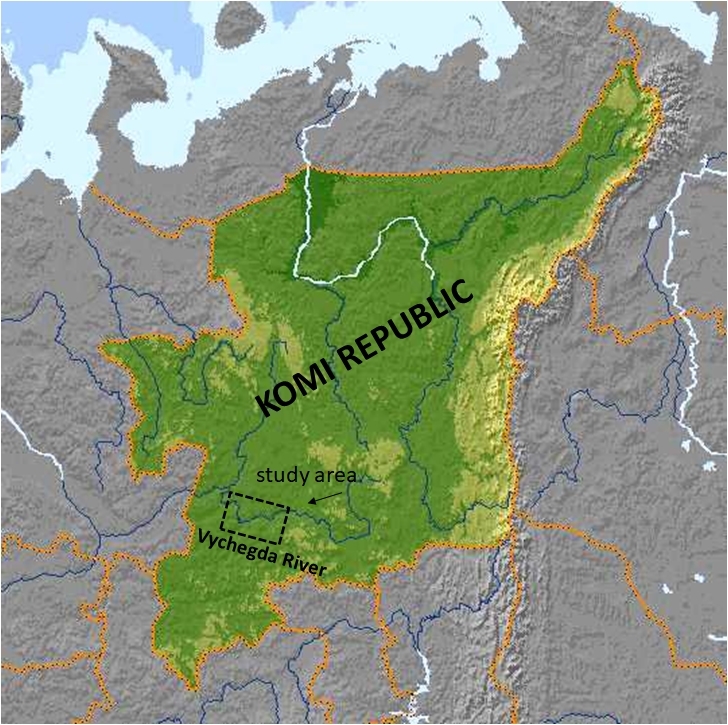
The map of the study area.

Our study area is located in the middle reaches of the river. The Middle Vychegda Basin occupies a vast valley; the floodplain is wide, usually bilateral, boggy, with numerous channels. Low-level, sloping shores alternate with high steep ones (3–18 m). The riverbed has a width of 100 to 680 m. There are many shallows and low alluvial sandy islands; the bottom of the river consists of sand, clay and pebbles. The current velocity is from 0.3–0.6 m/s (summer low water) to 1.5–1.8 m/s (high water) (Taskaev 1997). The ecosystem of the Vychegda River is experiencing a strong anthropogenic load as a result of municipal, agricultural and industrial effluents. In the waters of the river, a number of chemical elements and compounds exceed the Maximum Permissible Concentrations (MPC) (Kondratenok 2020). In some sections of the river, there is an increased temperature background (Elsakov and Shchanov 2016), which is the result of the inflow of wastewater from the sewage treatment facilities of the Mondi Syktyvkar JSC and the municipal wastewater of the City of Syktyvkar into the channel. The temperature increase over the background values is observed in the area over a distance up to 15 km from the source(Fig. 1).

The research was carried out in zone impacted by wastewaters of Mondi Syktyvkar JSC, in the middle reaches of the Vychegda River. On the section of the river (55 km long), seven sampling sites were selected: IB – background zone, above the zone of direct influence of wastewater from the pulp and paper industry (near Sedkyrkeshch Village), I – zone of direct influence of wastewater from the pulp and paper industry, II – 22.8 km lower than point I (near Kochchojyag Village), III – 6.4 km lower than point II (near Sluda Village), IV – zone of direct influence of wastewater from the pulp and paper industry (near Gavrilovka Village), V – 11.8 km lower than point IV (near Sotchem-vyv Village) and VI – 5.5 km lower than point V (near Ust'-Pozheg Village).

Throughout the period of our studies, weather conditions changed from year to year. For instance, in 2018 and 2020, the temperature indicators were similar: the average monthly air temperature in July reached +19.5°С and 20.0°С, respectively. In July 2019, the lowest average monthly temperature over the period of our work was registered: +15.4°C (with a deviation from the norm by -2.1°C). The water temperature in the studied section of the river varied in accordance with the air temperature. The minimum water temperature values were registered in July 2019: from 16.3°C to 17.1°C. In warmer 2018 and 2020, the July water temperature ranged from 21.8°C to 25.8°C and from 23.8°C to 24.7°C, respectively. Water levels at the time of sampling in 2018 and 2019 were similar. However, in 2018, sampling was conducted during the period of an intensive decrease in the water level, and, in 2019 – during the period of an intensive rise in the water level due to significant precipitation. In 2020, during the sampling period, the water level was significantly lower than in the previous two years (Patova et al. 2021). Our studies in 2018-2020 showed improvement of the quality of the river water in places of discharge of wastewater and changes in the environmental situation in connection with the modernisation of the treatment facilities of the Mondi Syktyvkar JSC (in 2014-2019). In 2020, concentrations of some ions in wastewater discharge sites: Cl-, SO42-, PO43-, NH4+, Stat, K, Na, phenol, as well as COD, permanganate value, turbidity and, electrical conductivity increased relative to background points (Patova et al. 2021).

### Design description

The Dataset (Baturina et al. 2021b) provides current data on aquatic invertebrates fauna (zooplankton and zoobenthos) of the Vychegda River (the Northern Dvina Basin) in the zone impacted by wastewaters from the pulp and paper industry (Mondi Syktyvkar JSC). The goal of the project is to prepare a basis for assessing and analysing changes in benthic invertebrate and plankton communities and species diversity of aquatic invertebrate, under the influence of treated wastewater from the pulp and paper production. The collection of hydrobiological samples was carried out mainly during the summer dry season. Standard hydrobiological methods were used. The data mainly concern the description of the species composition of each hydrobiological sample. Data include 23 taxonomic groups of zoobenthos and 152 lower-rank taxa for some groups: Oligochaeta, Mollusca, Cladocera, Copepoda, Coleoptera, Ephemeroptera, Plecoptera, Trichoptera and 120 taxa of zooplankton (Rotifera, Cladocera, Copepoda). A total of 8720 findings are included in the resource.

### Funding

The Ministry of Education and Science of the Russian Federation. Project No АААА-А17-117112850235-2 "Distribution, systematics and spatial organization of fauna and animals population in taiga and tundra landscapes and ecosystems at the Northeast European Russia"; agreement with Mondi Syktyvkar JSC № 45-2018/180405 "Assessment of long-term impact of Mondi Syktyvkar JSC on the biological diversity in the production area".

## Sampling methods

### Study extent

**Figure 2.**
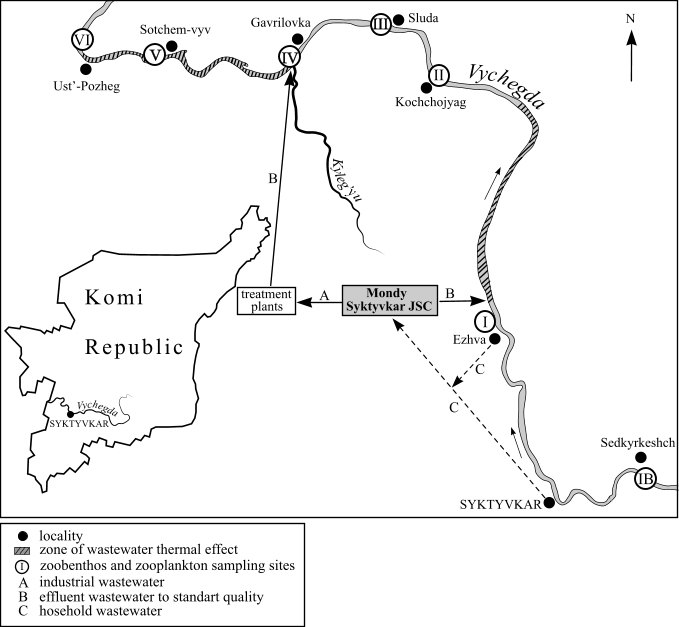
Figure 2. The map of the research area (from Patova et al. 2021).

(Fig. 2) The data paper is based on one dataset (8720 occurrences).

The dataset provides information on the number of individuals of aquatic invertebrates in zoobenthos samples and the number of individuals per cubic metre in zooplankton samples. Hydrobiological samples were collected on the part of the river located in the zone influenced by the pulp and paper industry (Mondi Syktyvkar JSC).

### Sampling description

The research was carried out in zone impacted by wastewaters of Mondi Syktyvkar JSC, in the middle reaches of the Vychegda River. The material includes data collected during the period of modernisation of the enterprise.

On the section of the river (55 km long), seven points were selected (Fig. 2), which are located at different distances from the wastewater inflow locations of wastewaters discharge of the enterprise (Mondi Syktyvkar JSC). At each point, 2-3 samples of zoobenthos and 2-3 samples of zooplankton were taken, from the right, left bank and middle of the river (if possible). The studies were carried out in July 2018-2020.

Zooplankton samples were collected using plankton nets with subsequent filtration through mesh nylon nets with 82-100 µm mesh size (Morduhaj-Boltovskoj 1975). Quantitative zoobenthic samples were taken from different depths (from 0.5 to 3.5 m).

For each point, a general description of the studied river was done and pH, mineralisation and temperature were measured. The identification of organisms was carried out according to the keys to freshwater invertebrates. More than 40 % of taxa (10 of model groups from 24 groups of zooplankton and zoobenthos) were determined up to the species level or genus.

Each sample was provided with a description that includes: collection date, locality (with geographic coordinates), device description, depth of sampling, water temperature, habitat (with substrate type, aquatic vegetation type, distance to the zone impacted by wastewaters from the Mondi Syktyvkar JSC), collector name, determined by (identification).

### Quality control

The data were collected and identified by specialists from the Institute of Biology of Komi Scientific Center of the Ural Branch of the Russian Academy of Sciences. Morphological analysis of specimens were performed using compound microscopes BIMAM R13-1 and Leica DM 4000B. Most of the Rotifera, Cladocera and Copepoda organisms from zooplankton samples and Oligochaeta, Cladocera, Copepoda, Coleoptera, Ephemeroptera, Plecoptera and Trichoptera from benthos samples were identified to the species level. The rest of the organisms from benthos samples were identified only as higher level taxa. For identification of species and higher-level taxa, we used both standards keys and data reported in modern studies specifically addressing the taxonomy of these groups (Rylov 1948, Chekanovskaja 1962, Lepneva 1964, Manujlova 1964, Smirnov 1971, Smirnov 1976, Kutikova 1977, Kutikova and Starobogatov 1977, Dumont and Pensaert 1983, Elliott et al. 1988, Lillehammer 1988, Koste and Shiel 1989, Boruckij et al. 1991, Shiel and Koste 1992, Tsalolikhin 1994, Nogrady et al. 1995, Segers 1995, Tsalolikhin 1995, De Smet 1996, De Smet 1997, Tsalolikhin 1997, Narchuk and Tumanov 2000, Tsalolikhin 2001, Nogrady and Segers 2002, Ueda and Reid 2003, Korovchinsky 2004, Starobogatov et al. 2004, Kotov and Stifter 2006, Teslenko and Zhiltzova 2009, Timm 2009, Alekseev 2010, Chertoprud and Chertoprud 2010, Bekker et al. 2012, Krivosheina 2012, Klimovsky and Kotov 2015, Tsalolikhin 2016, Wilke et al. 2019).

### Step description

The dataset included our own species list of plankton and benthos fauna of the Vychegda River, based on 63 zooplankton and zoobenthos samples collected in 2018-2020. Samples were taken by standard hydrobiological methods using the Petersen dredge (sampling area 0.025 m^2^), while those from shallow depths and on rocky bottoms (i.e. gravel) were collected by a hydrobiological scraper (0.09 m^2^) with mesh size ≤158 μm (Zinchenko 2014) for zoobenthosand a net with mesh nylon nets (82–100 μm) for zooplankton (Morduhaj-Boltovskoj 1975). Samples were preserved in 4% formaldehyde (in the field) and examined under light microscopes in the laboratory. Further identification of aquatic invertebrates was carried out in laboratory conditions. The identification of species of invertebrates was carried out with the preparation of temporary or permanent specimens, under a microscope using keys to identify each taxonomic group. For each sample, the following were described: collection date, locality (with geographic coordinates), device description, sampling depth, water temperature, habitat (with substrate type, aquatic vegetation type, distance to the zone impacted by wastewaters from the Mondi Syktyvkar JSC), collector name, identificator name. For some species, permanent preparations have been made. Design of sampling was based on the regular arrangement. The material includes data collected during the period of modernisation and reduction of emissions of the enterprise.

## Geographic coverage

### Description

The studied area is located in the middle part of the Vychegda River (Еuropean North-East of Russia, Komi Republic) (Fig. 1). The Vychegda is the largest right tributary of the Northern Dvina River. The study was carried out in the areas impacted by wastewaters from the Mondi Syktyvkar JSC, which is the largest pulp and paper industry enterprise in the European part of Russia.

On the section of the river (55 km long), seven points were selected (Fig. 2): IB – background zone, above the zone directly influenced bywastewaters from the pulp and paper industry, I – zone directly influenced by wastewater from the pulp and paper industry, II – 22.8 km lower than point I, III – 6.4 km lower than point II, IV – zone directly influenced by wastewater from the pulp and paper industry, V – 11.8 km lower than point IV, VI – 5.5 km lower than point V.

The dataset includes data on aquatic invertebrates from 40 zoobenthos samples and 23 zooplankton samples.

## Taxonomic coverage

### Description

The dataset contains information obtained from sampling for aquatic invertebrates (zooplankton and zoobenthos) (Table 1). Some invertebrates were identified from type to species or taxa (subspecies) of a lower rank. The dataset includes detailed information: 1. taxa (family, genus, species, subspecies) of some (model) groups of zoobenthos: Oligochaeta (1720 occurrences), Mollusca (161), Cladocera (680), Copepoda (680), Coleoptera (160) , Ephemeroptera (1120), Plecoptera (240), Trichoptera (480) and Diptera (360); 2. taxa of the highest rank of zoobenthos groups: Hydrozoa, Nematoda, Hirudinea, Ostracoda, Tardigrada, Hydrachnidia, Araneae, Collembola, Hemiptera, Megaloptera and Odonata (440 occurrences); 3. taxa (species, subspecies) of zooplankton: Rotifera (1638), Cladocera (762) and Copepoda (279). A total of 8720 occurrences are included in the resource. In the dataset, full taxonomic affiliation is given for each record, including: Type, Class, Subclass, Order, Family, Subfamily, Genus, Subgenus, Species, lifestage, individuals per sample (for zoobenthos) and individuals per m^3^ (for zooplankton).

Our study showed how wastewater from a pulp and paper mill can affect aquatic invertebrates. For this purpose, we chose six sampling sites in the middle reaches of the Vychegda River, in the zone affected by the treated wastewater of the Mondi Syktyvkar JSC at different distances from the wastewater discharge points. According to the state of invertebrate communities in general, the river waters in the studied area can be classified as "oligotrophic" (in terms of zooplankton) or "satisfactory" (in terms of zoobenthos). The exception is the sites located downstream of the wastewater discharge points that are classified as "eutrophic" (in terms of zooplankton) and the treated wastewater discharge points which are classified as "unsatisfactory" (in terms of zoobenthos). However, given the high indicators of the quantitative development of aquatic invertebrate communities and their high species diversity, we can say that there is no toxic effect of wastewater in the studied area, but there are processes of anthropogenic eutrophication of the river.

Analysis of the species composition of model groups – microcrustaceans, oligochaetes, non-chironomid amphibiotic insects – revealed a sufficiently high level of community diversity. The diversity in the groups varied depending on the sampling site, but the differences in values for most of the sites were insignificant. The greatest diversity for all groups was observed at points III and V.

Amongst all the diversity of species of aquatic invertebrates found in the investigated section of the Vychegda River, we noted new species for the regional fauna, *Elaphoidellabidens* (Schmeil, 1983) and *Moinamacrocopa* (Straus, 1820), as well as a rare species, *Brachycercusharisella* (Curtis, 1834), listed in the Red Book of the Komi Republic.

## Temporal coverage

### Notes

Data sources provided the dates when the species was detected for the first time in the zone impacted by wastewaters from the pulp and paper enterprise of the 8720 occurrences included in the dataset. The earliest first record dates back to 2018 and the most recent event occurred in 2020.

Field studies of zoobenthos were carried out on 23, 24 July 2018, 13-17 July 2019 and 20, 21 July 2020. At each point, from two to five samples were taken. In points I and IV was carried out annually, in item III - in 2018 and 2019, the rest were collected once.

## Usage licence

### Usage licence

Creative Commons Public Domain Waiver (CC-Zero)

### IP rights notes

IP rights notes: This work is licensed under a Creative Commons Attribution (CC-BY) 4.0

License.

## Data resources

### Data package title

The fauna of aquatic invertebrates in the river impacted by wastewaters from the pulp and paper industry (Komi Republic)

### Resource link

https://www.gbif.org/dataset/5a6c4b09-bd03-4a8a-b673-4a5d5430eea2

### Alternative identifiers

5a6c4b09-bd03-4a8a-b673-4a5d5430eea2, http://ib.komisc.ru:8088/ipt/resource?r=cop

### Number of data sets

1

### Data set 1.

#### Data set name

The fauna of aquatic invertebrates in the river impacted by wastewaters from the pulp and paper industry (Komi Republic)

#### Data format

Darwin Core Archive format

#### Number of columns

44

#### Character set

UTF-8

#### Download URL

5a6c4b09-bd03-4a8a-b673-4a5d5430eea2, http://ib.komisc.ru:8088/ipt/resource?r=cop

#### Description

The dataset includes two tables related by the eventID field – Events and Associated occurrences

**Data set 1.** 

Column label	Column description
eventID (Event Core)	An identifier for the event (layer).
eventDate (Event Core)	The date-time or interval during which an Event occurred.
year (Event Core)	The year in which the Event occurred, according to the Common Era Calendar.
month (Event Core)	The month in which the Event occurred.
day (Event Core)	The day in which the Event occurred.
habitat (Event Core)	A category or description of the habitat in which the Event occurred.
verbatimDepth (Event Core)	The original description of the depth below the local surface.
eventRemarks (Event Core)	Comments or notes about the Event.
samplingProtocol (Event Core)	The descriptions of the methods used during an Event.
sampleSizeValue (Event Core)	A numeric value for a measurement of the size of a sample in a sampling event.
sampleSizeUnit (Event Core)	The unit of measurement of the size (time duration, length, area or volume) of a sample in a sampling event.
locationID (Event Core)	An identifier for the set of location information.
continent (Event Core)	The name of the continent in which the Location occurs.
country (Event Core)	The name of the country in which the Location occurs.
countryCode (Event Core)	The standard code for the country in which the Location occurs.
stateProvince (Event Core)	The name of the next smaller administrative region than country (republic) in which the Location occurs.
locality (Event Core)	The specific description of the place.
locationRemarks (Event Core)	Comments or notes about the Location.
decimalLatitude (Event Core)	The geographic latitude.
decimalLongitude (Event Core)	The geographic longitude
geodeticDatum (Event Core)	The ellipsoid, geodetic datum or spatial reference system (SRS) upon which the geographic coordinates given in decimalLatitude and decimalLongitude are based.
georeferencedBy (Event Core)	A list of names of people who determined the georeference (spatial representation) for the Location.
coordinateUncertaintyInMetres (Event Core)	The horizontal distance (in metres) from the given decimalLatitude and decimalLongitude describing the smallest circle containing the whole of the Location.
occurrenceID (Occurrence Extension)	An identifier for the Occurrence.
basisOfRecord (Occurrence Extension)	The nature of the related resource.
Phylum (Occurrence Extension)	The full scientific name of the phylum or division in which the taxon is classified.
Class (Occurrence Extension)	The full scientific name of the class in which the taxon is classified.
order (Occurrence Extension)	The full scientific name of the order in which the taxon is classified.
family (Occurrence Extension)	The full scientific name of the family in which the taxon is classified.
genus (Occurrence Extension)	The full scientific name of the genus in which the taxon is classified.
specificEpithet (Occurrence Extension)	The name of the first or species epithet of the scientificName.
infraspecificEpithet (Occurrence Extension)	The name of the lowest or terminal infraspecific epithet of the scientificName, excluding any rank designation.
identificationQualifier (Occurrence Extension)	A brief phrase or a standard term ("cf.", "aff.") to express the determiner's doubts about the Identification.
scientificNameAuthorship (Occurrence Extension)	The authorship information for the scientificName formatted according to the conventions of the applicable nomenclaturalCode.
scientificName (Occurrence Extension)	The full scientific name, with authorship and date information, if known.
organismRemarks (Occurrence Extension)	Comments or notes about the Organism instance.
taxonRank (Occurrence Extension)	The taxonomic rank of the most specific name in the scientificName.
Lifestage (Occurrence Extension)	The age class or life stage of the Organism(s) at the time the Occurrence was recorded.
organismQuantityType (Occurrence Extension)	The type of quantification system used for the quantity of organisms.
individualCount (Occurrence Extension)	The number of individuals present at the time of the Occurrence.
occurrenceStatus (Occurrence Extension)	A statement about the presence or absence of a Taxon at a Location.
recordedBy (Occurrence Extension)	A list of names of people responsible for recording the original Occurrence.
identifiedBy (Occurrence Extension)	A list of names of people who assigned the Taxon to the subject.
associatedReferences (OccurrenceExtension)	A list of publication of literature associated with the Occurrence.

## Figures and Tables

**Table 1.** List of taxa.

Rank	Scientific name	Common name
phylum	Cnidaria	
class	Hydrozoa	hydras
phylum	Nematoda	nematodes
Phylum	Rotifera	rotifers
class	Eurotatoria	
subclass	Bdelloidea	
order	Bdelloida	
subclass	Monogononta	
order	Ploima	
order	Flosculariacea	
phylum	Tardigrada	tardigrades
phylum	Annelida	
class	Clitellata	
subclass	Oligichaeta	oligochaetes
order	Tubificida	
order	Lumbriculida	
order	Enchytraeida	
subclass	Hirudinea	leeches
phylum	Mollusca	shellfish
class	Gastropoda	
phylum	Arthropoda	
class	Branchiopoda	
subclass	Diplostraca	
order	Anomopoda	
order	Ctenopoda	
order	Onychopoda	
order	Haplopoda	
class	Hexanauplia	
subclass	Copepoda	copepods
order	Cyclopoida	
order	Harpacticoida	
class	Ostracoda	ostracods
class	Hydrachnidia	water mites
class	Arachnida	
order	Araneae	spiders
class	Collembola	springtail
class	Insecta	insects
order	Hemiptera	bedbugs
subclass	Pterygota	
order	Ephemeroptera	mayflies
order	Plecoptera	stoneflies
order	Megaloptera	alderflies
order	Coleoptera	beetles
order	Trichoptera	caddisflies
order	Odonata	dragonflies
order	Diptera	flies
